# Supplementary figures and images for: Multi-Material 3D Printing of Biobased Epoxy Resins
Source: Polymers (Basel). 2024 May 27;16(11):1510. doi: 10.3390/polym16111510 (PMC11174478; doi:10.3390/polym16111510)

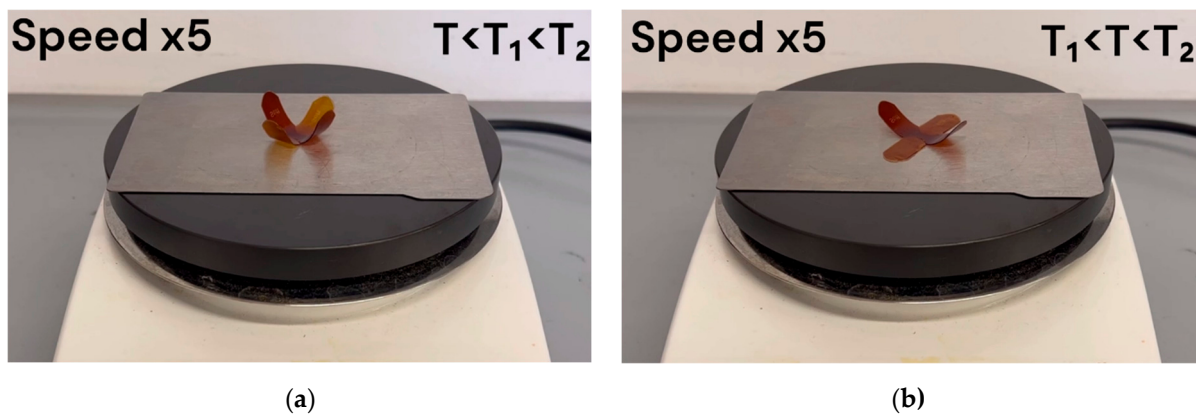

**Figure S1.** Different shape of the printed flower at different temperatures.

Supplement: Supplementary file 1 [file polymers-16-01510-s001.zip › polymers-3005738-supplementary.pdf]
